# Research on cooperative advertising strategies for dual channel supply chain of fresh agricultural products considering carbon reduction efficiency under retailer leadership

**DOI:** 10.1371/journal.pone.0303525

**Published:** 2024-06-12

**Authors:** Wenbo Wang, Aimin Zhu, Lijuan Yu, Hongjiang Wer

**Affiliations:** School of Management, Shenyang University of Technology, Shenyang, PR China; Shandong University of Science and Technology, CHINA

## Abstract

With the development of low-carbon economy and the dominant position of retailers, through the establishment and comparison of three cooperative advertising models: model of supplier’s independent advertising, model of supplier’s independent advertising and model of retailer’s and supplier’s cooperative advertising, this paper studies the cooperative advertising decision-making of fresh agricultural products supply chain with two channels, and the demand of both channels is affected by the level of advertising investment, the proportion of advertising cost sharing and the efficiency of carbon emission reduction. The results show that when supplier and retailer adopt the two-way cooperative advertising mode, the demand and profit level of the two channels reach the optimal level. Numerical analysis shows that carbon emission reduction efficiency has an important impact on profits and market demand, which is closely related to cooperative advertising strategy.

## 1 Introduction

With the frequent occurrence of global disastrous weather, the development of low-carbon economy to cope with the impact of global warming has become a global consensus [1, 2]. Countries around the world have promoted the development of carbon emission reduction by issuing relevant policies and regulations [3–6], effectively improving consumers’ low-carbon awareness and increasing the sales of low-carbon goods. This also enables suppliers to increase investment in low-carbon operation and production management. Among the anthropogenic greenhouse gas emissions, the agricultural system accounts for more than one third [7], so it is urgent to vigorously reduce the carbon emissions of the agricultural products industry. As a large country producing and selling fresh agricultural products, China is also one of the largest carbon emission countries in the world [8]. At the same time, fresh agricultural products have the characteristics of easy deterioration and seasonality [9–12], their preservation has become a difficult problem in the process of production, transportation and sales [13, 14]. With the intensification of the national market competition and the improvement of low-carbon awareness, the fresh agricultural products industry urgently needs to expand the low-carbon market, so reducing carbon emissions and expanding the market demand for fresh agricultural products is one of the issues worthy of in-depth research [15, 16].

Under certain conditions, consumers are more eager to buy “low-carbon products” [17, 18]. However, low-carbon fresh agricultural products still have problems such as high prices and difficult marketing. With the rapid development of the Internet and the gradual improvement of the logistics network, online shopping has become an important shopping method for consumers [19]. At present, more and more suppliers have launched online direct sales channels, through the integration of online and offline channels for consumers to obtain a better shopping experience [20]. Previous studies have shown that when suppliers both supply for retailers and compete with retailers, it will definitely cause competiton between supply chains at the same level. In order to solve the conflict between channels, suppliers and retailers introduce cooperative advertising to weaken the negative impact of competition between the two sides [21]. In cooperative advertising, there are three situations: one-way investment by suppliers or retailers, and joint investment by both parties. Participants will bear a certain proportion of the advertising costs of the other party, which is called the participation rate [21, 22]. Although cooperative advertising strategy occupies an important position in economic activities, blind investment in cooperative advertising may lead to a decline in the profits of various stakeholders [23, 24]. Vendor-initiated online advertising, increased market demand through vertical advertising partnerships, and customers who have viewed online ads are more likely to make purchases in offline stores. At the same time, retailers place offline promotional ads and other advertisements and receive support from suppliers, effectively promoting the brand and making it possible for consumers to shop for the brand at home through online channels, increasing convenience [24]. With the vigorous development of the customer-oriented market economy, retailers’ voice in the supply chain is gradually increasing, and in actual production and life, there are many cases where retailers occupy a dominant position. Therefore, the research on the low-carbon development and cooperative advertising strategy of the dual channel supply chain of fresh agricultural products under the leadership of retailers has important scientific research and practical application value.

This paper aims to solve the problem of cooperative advertising strategy in the dual channel supply chain of fresh agricultural products under the low-carbon background. The structure of this paper is as follows. Section 2 provides a literature review of the dual channel supply chain of fresh agricultural products, carbon emission reduction technologies and cooperative advertising strategies. In Section 3, the research problems in this paper are described and the relevant symbols are defined. In Section 4, the mathematical model proposed in this study is constructed and solved. In Section 5, we analyzethe mathematical model by calculating the relationship between the level of advertising investment, the share ratio and the prize. In Section 6, the numerical analysis of the model proposed in this paper is carried out to verify and supplement the theoretical model in this paper. The last section summarizes the research and provide implications and future research prospects.

## 2 Literature review

### 2.1 Research on dual channel supply chain of fresh agricultural products

In the process of studying the fresh agricultural products supply chain, many scholars have early studied the single channel supply chain mode of supplier supply and retailer sales [25–27]. With the rapid development of technology and the Internet, e-commerce has shown a blowout trend, and online shopping has become the main shopping mode of consumers [28, 29]. However, due to the perishable nature of fresh agricultural products and the poor accessibility of online shopping, the e-commerce market for fresh agricultural products still has a large expansion space [20]. The study on the dual channel supply chain of fresh agricultural products mainly focuses on pricing and inventory decisions. For example, Dan et al. [30] investigate the optimal combination of pricing and retailing services through the stackelberg game approach by establishing the manufacturer, retailer and dual-channel supply chain profit functions in decentralized and centralized models, respectively; Hu et al. [31] establish four Stackelberg game models to provide optimization methods for agricultural supply chains from a green development perspective; Zhang et al. [32] used stackelberg game to study the POPU strategy (Preorder-online, pickup-in-store) under competitive situations, comparing the retailers’ sales strategy choices under decentralized and centralized decision-making, which can effectively increase the market share and profitability when the POPU operation cost is lower; Zheng et al. [33] considered the quality loss and quantity loss at the same time, and built the mathematical models of single-stage and multi-stage discount strategies respectively. By comparing the optimal discount ratio under decentralized decision making and centralized decision making models, they coordinated the dual channel supply chain. It can be seen that the dual channel sales model combining online and offline sales is favored by many suppliers, but the introduction of new channels is bound to have an impact on the original traditional channels, so Yan et al. [34] introduced a shared contract to ease the conflict between the two channels and further promote the development of the dual channel supply chain of fresh agricultural products.

### 2.2 Research on carbon emission reduction and cooperative advertising in the supply chain

In recent years, the problem of global warming has attracted the attention of countries all over the world, and all trades and professions have developed and applied carbon emission reduction technologies to deal with the environmental crisis [35, 36]. According to the report of the road to agricultural carbon neutrality, the greenhouse gases emitted by agricultural products in the whole life cycle account for 21% to 37% of the total social output [37]. Therefore, the introduction of low-carbon fresh agricultural products is of great significance for increasing sustainable agricultural consumption, promoting its transformation to green and reducing greenhouse gas emissions [26, 37]. At present, the application of carbon emission reduction technology in fresh agricultural products industry is less, and investment in carbon emission reduction technology will increase the difficulty of commodity production and sales. Previous studies have shown that cooperative advertising can effectively improve product sales [38]. Therefore, under the background of carbon emission reduction, how to choose the appropriate cooperative advertising strategy has become one of the research hotspots. The research on cooperative advertising strategy can be traced back to 1972 [39]. On this basis, Li et al. [40] explored the influence of brand effect, local advertising and sharing policy on cooperative advertising strategy, and built three game models to analyze the best marketing strategy of manufacturers and suppliers. As retailers have a better understanding of the local market and lower advertising costs, it is effective to strengthen advertising cooperation between suppliers and retailers [41]. As we all know, the main influencing factors of cooperative advertising strategy are: cooperation mode, subsidy rate, promotion strength, etc. [42], and the Stackelberg game method is also widely used in various fields of supply chain research, so many scholars have used the Stackelberg game method to determine the optimal cooperative advertising strategy [43–45]. Karray et al. [46] made an in-depth comparison of the impact of cooperative advertising on suppliers’ profits, and provided practical help for suppliers’ choice of cooperative advertising strategies.

### 2.3 Research gap

The application summary in relevant literature is shown in Table 1. In general, with the increasing status of retailers in the supply chain, under certain circumstances, retailers have the ability and opportunity to dominate the supply chain [47]. In addition, study on carbon emission reduction decision and dual channel supply chain of fresh agricultural products industry has reached some conclusions [27, 35, 48–50]. However, due to the active investment in the development of carbon emission reduction in the fresh agricultural products industry, the profit decline caused by the increase of production costs of suppliers, and the poor sales caused by the low degree of commodity recognition need to be solved urgently. Therefore, choosing appropriate ways to help the fresh agricultural products industry improve market demand and increase the profits of all parties has become a research hotspot. At present, the study on carbon emission reduction of fresh agricultural products supply chain and cooperative advertising strategy is still in the initial stage, and cooperative advertising strategy has shown a positive effect on improving the profit level of participants in the supply chain [22]. Therefore, whether the cooperative advertising strategy for the dual channel supply chain of fresh agricultural products can get out of the dilemma is worth further study. And because the supply chain is developing in the direction of diversification, which increases the discourse power of retailers. In view of this, this paper takes the dual channel supply chain of fresh agricultural products composed of a supplier with online direct sales ability and a retailer with offline sales ability as the research object. The retailer plays a leading role in the supply chain system, while the supplier actively responds to the call of the state to produce and sell low-carbon fresh agricultural products, and cooperates with the retailer to promote the goods to consumers by using cooperative advertising strategy. Under the background of carbon emission reduction, the selection of cooperative advertising strategies between suppliers and retailers, as well as the optimal level of cooperative investment, and the relationship between the influencing factors are solved by numerical analysis. Through this study, the existing literature is supplemented.

**Table 1:** Summary of the scenarios in the related literature.

Author	Supply Chain System	Fresh Agricutural product	carbon emission	cooperative advertising
Gao et al. [15]	Single channel supply chain	Y	N	N
Yang and Yao [16]	Single channel supply chain	Y	N	N
Dye [27]	Single channel supply chain	Y	N	N
Yan et al. [34]	Dual channel supply chain	Y	N	N
Qing [43]	Single channel supply chain	N	N	Y
Xu et al. [50]	Dual channel supply chain	Y	Y	N
This paper	Dual channel supply chain	Y	Y	Y

## 3 Problems and symbols

**Fig 1:**
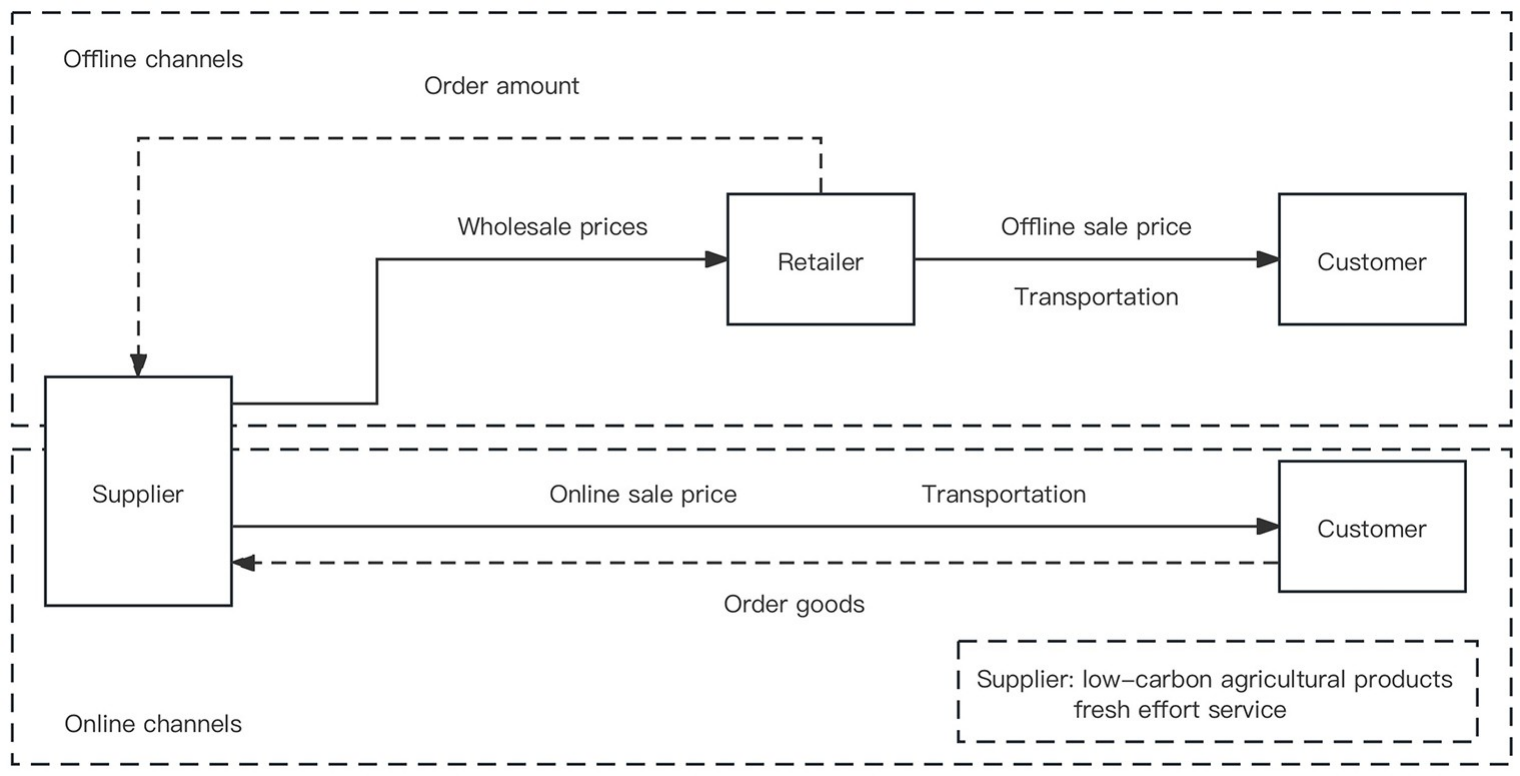
Fresh agricultural products dual channel supply chain model diagram.

Under the low-carbon background, the cooperative advertising strategy of fresh agricultural products in a dual channel supply chain dominated by retailers are studied in the paper. The sales channels are divided into: ① Supplier sell products to consumers through online direct sales channels; ② Supplier wholesales products to retailer, who sells products to customers through traditional offline channels. We research the fresh agricultural products dual channel supply chain model is shown in Fig 1.

By introducing the level of advertising investment and the proportion of advertising cost sharing, we constructs the profit model of supplier and retailer to find its equilibrium solution, and obtains the optimal profits of each party under three different cooperative advertising strategies: one-way investment by suppliers, one-way investment by retailers and two-way investment by both parties, and analyzes the impact of carbon emission reduction efficiency on cooperative advertising strategy and the profit level of various stakeholders. Three cooperative advertising strategies are considered in this paper. The subscripts *m*, *r* and *mr* are used to represent suppliers, retailers and the whole supply chain, respectively. The superscript “*RM*” represents the one-way advertising mode of suppliers, “*RR*” represents the one-way advertising mode of retailers, and “*RC*” represents the cooperative advertising mode of suppliers and retailers. In the process of solving, the superscript “*” is used to represent the optimal decision variable. The symbol interpretation of other relevant variables in the text is shown in Table 2.

**Table 2:** Description of relevant parameters and symbols.

Symbols	Illustrate
*T*	Total scale of market demand
*θ*	Consumer acceptance of supplier’s online direct sales channel
*η*	Impact ratio of supplier’s online advertising investment on offline demand
*γ*	Impact ratio of retailer’s offline advertising investment on online demand
*π*_*m*_	Supplie’s profit
*π*_*r*_	Retalier’s profit
*π*_*mr*_	Supply chain’s profit
*D*_*m*_	Online demand
*D*_*r*_	Offiine demand
*A*_*m*_/*A*_*r*_	Advertising investment level of supplier / retailer
*K*_*m*_/*K*_*r*_	Influence coefficient of advertising investment level of supplier / retailer on market demand
*t*_*m*_	The proportion that the manufacturer is willing to bear when the retailer invests in advertising
*t*_*r*_	The proportion that retailers is willing to bear when supplier invests in advertising
*p*_*m*_	Supplier online sales price
*p*_*r*_	Retail sales price
*ω*	Wholesale price per unit product
*c*	Production cost per unit of goods *c* < *ω* < *p*_*m*_ < *p*_*r*_
*α*	Carbon reduction efficiency of supplier unit products
*μ*	Sensitivity coefficient of market demand to carbon reduction efficiency per unit product
*δ*	Cost premium coefficient for carbon reduction per unit product (supplier)
*β*	Supplier’s efforts in product preservation
λ	Sensitivity coefficient of market demand to unit product preservation efforts
*φ*	Cost premium coefficient for unit product preservation effort (supplier)

To simplify the above issues, we make the following assumptions:

Assumption 1: Assuming that supplier and retailer are in a situation of information completion symmetry, and both rely on maximizing their own interests as the basis for decision-making;

Assumption 2: Assuming that supplier invests in carbon reduction technologies under the preference of consumers with low-carbon awareness, and retailer doesn’t participate in the research and application costs of carbon reduction technologies, but is willing to increase the sales of low-carbon fresh agricultural products through cooperative advertising;

Assumption 3: Due to the uncertainty of cooperative advertising on channel sales, we only consider the impact of cooperative advertising on product sales, without considering its impact on pricing, and assumes that the cost function of cooperative advertising is $C\left(A\right)=\frac{A_{i}^{2}}{2},i=m/r$ [42, 43].

## 4 Model construction

**Fig 2:**
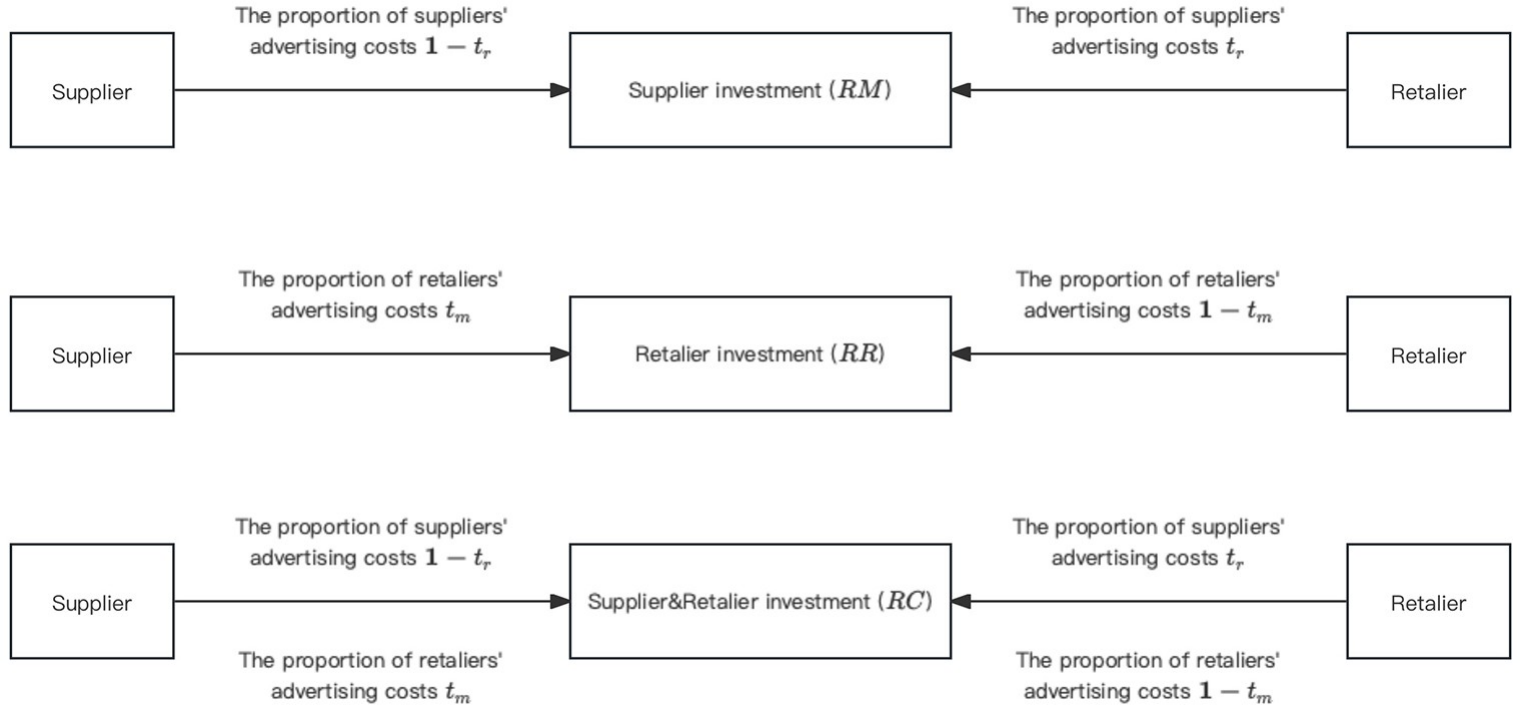
Schematic diagram of cooperative advertising model.

According to the existing research, the promotion of low-carbon products can help to improve sales. This paper uses the research method of literature [38] to divide the cooperative advertising mode into the following three cases: *RM* (Advertising by supplier), *RR* (Advertising by retalier) and *RC* (Advertising by both supplier and retalier). Among the three cooperative advertising models, we aim to maximize the profits of supply chain participants. The three cooperative advertising modes are shown in Fig 2.

### 4.1 Model of supplier’s independent advertising (*RM* model)

In *RM* model, the supplier invests in cooperative advertising in one direction, and the retailer takes the initiative to bear a certain proportion of cooperative advertising costs. According to the above questions and symbols, and with reference [38], the demand function and profit function of supplier’s online direct sales channels and retailer’s offline sales channels are described in this section.

Supplier online channel demand function is as follows:

$$\begin{array}{c} D_{m}^{RM}=\theta \left(T+\mu \alpha +\lambda \beta \right)+\eta K_{m}A_{m}, \end{array}$$ (1)

Retailer offline channel channel function is as follows:

$$\begin{array}{c} D_{r}^{RM}=\left(1-\theta \right)\left(T+\mu \alpha +\lambda \beta \right)+\left(1-\eta \right)K_{m}A_{m}, \end{array}$$ (2)

Supplier’s profit function is as follows:

$$\begin{array}{c} \pi_{m}^{RM}=\left(\omega -c-\alpha \delta \right)D_{r}^{RM}+\left(p_{m}-c-\alpha \delta \right)D_{m}^{RM}-\frac{\varphi }{2}\beta^{2}-\frac{1-t_{r}}{2}A_{m}^{2}, \end{array}$$ (3)

Retalier’s profit function is as follows:

$$\begin{array}{c} \pi_{r}^{RM}=\left(p_{r}-\omega \right)D_{r}^{RM}-\frac{t_{r}}{2}A_{m}^{2}, \end{array}$$ (4)

Supply chain’s profit function is as follows:

$$\begin{array}{c} \pi_{mr}^{RM}=\left(p_{r}-c-\alpha \delta \right)D_{r}^{RM}+\left(p_{m}-c-\alpha \delta \right)D_{m}^{RM}-\frac{\varphi }{2}\beta^{2}-\frac{A_{m}^{2}}{2}, \end{array}$$ (5)

According to the demand and profit function of each participant in the supply chain and the Stackelberg game order, theorem 1 can be obtained.

**Theorem 1:** In this case, according to stackelberg game, the supplier determines the advertising level first, we find the partial derivative of advertising investment level in the supplier profit function:

$$\begin{array}{c} \frac{\partial \pi_{m}^{RM}}{\partial A_{m}}=\left(1-\eta \right)\left(\omega -c-\alpha \delta \right)K_{m}+\eta \left(p_{m}-c-\alpha \delta \right)K_{m}-\left(1-t_{r}\right)A_{m}. \end{array}$$ (6)

Make this function equal to 0, we can get the optimal advertising investment level from supplier is:

$$\begin{array}{c} A_{m}=\frac{\left(\eta p_{m}-\eta \omega +\omega -c-\alpha \delta \right)K_{m}}{1-t_{r}}. \end{array}$$ (7)

Then retailer determines the sharing proportion of cooperative advertising, we use the reverse induction method, the advertising level is regarded as a constant to determine the retailer’s sharing proprtion. By deriving the retailer’s profit function:

$$\begin{array}{c} \begin{array}{cc} \frac{\partial \pi_{r}^{RM}}{\partial t_{r}}= & \alpha \delta +c-\eta p_{m}-2\eta p_{r}+3\eta \omega +2p_{r}-3\omega +(\alpha \delta \\ + & c-\eta p_{m}+2\eta p_{r}-\eta \omega -2p_{r}+\omega )t_{r}. \end{array} \end{array}$$ (8)

Make this function equal to 0, avaliable optimal proportion of retailer is:

$$\begin{array}{c} t_{r}^{*}=\frac{\eta \left(p_{m}-\omega +2\left(p_{r}-\omega \right)\right)-2\left(p_{r}-\omega \right)+\omega -c-\alpha \delta }{\eta \left(\omega -p_{m}+2\left(p_{r}-\omega \right)\right)-2\left(p_{r}-\omega \right)-\left(\omega -c-\alpha \delta \right)}. \end{array}$$ (9)

Substituting $t_{r}^{*}$ into *A*_*m*_, we can get $A_{m}^{*}$:

$$\begin{array}{c} A_{m}^{*}=\frac{\left(\eta p_{m}-\eta \omega +\omega -c-\alpha \delta \right)K_{m}}{1-t_{r}}=\frac{K_{m}}{2}\left(\eta \left(p_{m}-2p_{r}+\omega \right)+2p_{r}-\omega -c-\alpha \delta \right). \end{array}$$ (10)

### 4.2 Model of retailers’ independent advertising (*RR* model)

In the *RR* model, this section lists the demand function and profit function of supplier and retailer.

Supplier online channel demand function is as follows:

$$\begin{array}{c} D_{m}^{RR}=\theta \left(T+\mu \alpha +\lambda \beta \right)+\gamma K_{r}A_{r}, \end{array}$$ (11)

Retailer offline channel channel function is as follows:

$$\begin{array}{c} D_{r}^{RR}=\left(1-\theta \right)\left(T+\mu \alpha +\lambda \beta \right)+\left(1-\gamma \right)K_{r}A_{r}, \end{array}$$ (12)

Supplier’s profit function is as follows:

$$\begin{array}{c} \pi_{m}^{RR}=\left(\omega -c-\alpha \delta \right)D_{r}^{RR}+\left(p_{m}-c-\alpha \delta \right)D_{m}^{RR}-\frac{\varphi }{2}\beta^{2}-\frac{t_{m}}{2}A_{r}^{2}, \end{array}$$ (13)

Retalier’s profit function is as follows:

$$\begin{array}{c} \pi_{r}^{RR}=\left(p_{r}-\omega \right)D_{r}^{RR}-\frac{1-t_{m}}{2}A_{r}^{2}, \end{array}$$ (14)

Supply chain’s profit function is as follows:

$$\begin{array}{c} \pi_{mr}^{RR}=\left(p_{r}-c-\alpha \delta \right)D_{r}^{RR}+\left(p_{m}-c-\alpha \delta \right)D_{m}^{RR}-\frac{\varphi }{2}\beta^{2}-\frac{A_{r}^{2}}{2}, \end{array}$$ (15)

According to the demand and profit function of each participant in the supply chain and the Stackelberg game order, theorem 2 can be obtained.

**Theorem 2:** In this section, according to stackelberg game, the supplier determines the proportion to be borne, we can get the partial derivative of the advertising allocation proportion in the supplier profit function:

$$\begin{array}{c} \frac{\partial \pi_{m}^{RM}}{\partial t_{m}}=-\frac{A_{r}^{2}}{2}. \end{array}$$ (16)

Avaliable optimal allocation proportion of supplier is:

$$\begin{array}{c} t_{m}^{*}=0. \end{array}$$ (17)

Then according to reverse method, retailer’s advertising investment is determined by taking supplier’s proportion as a constant. The calculation process is as follows:

$$\begin{array}{c} \frac{\partial \pi_{r}^{RR}}{\partial A_{r}}=\left(p_{r}-\omega \right)\left(1-\gamma \right)K_{r}-\left(1-t_{m}\right)A_{r}. \end{array}$$ (18)

Make this function equal to 0, we can get the optimal of retailer’s advertising investment level is:

$$\begin{array}{c} A_{r}=\frac{\left(p_{r}-\omega \right)\left(1-\gamma \right)K_{r}}{1-t_{m}}. \end{array}$$ (19)

Substituting $t_{m}^{*}$ into *A*_*r*_, we can get $A_{r}^{*}$:

$$\begin{array}{c} A_{r}^{*}=\frac{\left(p_{r}-\omega \right)\left(1-\gamma \right)K_{r}}{1-t_{m}}=\left(p_{r}-\omega \right)\left(1-\gamma \right)K_{r}. \end{array}$$ (20)

### 4.3 Model of retailer’s and supplier’s cooperative advertising (*RC* model)

In the *RC* model, the supplier and the retailer both invest in advertisements, and the proportion of the supplier bearing the retailer’s advertising cost is *t*_*m*_, and the proportion of the retailer bearing the supplier’s advertising cost is *t*_*r*_. Therefore, this section lists the demand function and profit function between the supplier and the retailer.

Supplier online channel demand function is as follows:

$$\begin{array}{c} D_{m}^{RC}=\theta \left(T+\mu \alpha +\lambda \beta \right)+\eta K_{m}A_{m}+\gamma K_{r}A_{r}, \end{array}$$ (21)

Retailer online channel demand function is as follows:

$$\begin{array}{c} D_{r}^{RC}=\left(1-\theta \right)\left(T+\mu \alpha +\lambda \beta \right)+\left(1-\eta \right)K_{m}A_{m}+\left(1-\gamma \right)K_{r}A_{r}, \end{array}$$ (22)

Supplier’s profit function is as follows:

$$\begin{array}{c} \pi_{m}^{RC}=\left(\omega -c-\alpha \delta \right)D_{r}^{RC}+\left(p_{m}-c-\alpha \delta \right)D_{m}^{RC}-\frac{\varphi }{2}\beta^{2}-\frac{1-t_{r}}{2}A_{m}^{2}-\frac{t_{m}}{2}A_{r}^{2}, \end{array}$$ (23)

Retailer’s profit function is as follows:

$$\begin{array}{c} \pi_{r}^{RC}=\left(p_{r}-\omega \right)D_{r}^{RC}-\frac{t_{r}}{2}A_{m}^{2}-\frac{1-t_{m}}{2}A_{r}^{2}, \end{array}$$ (24)

Supply chain’s profit function is as follows:

$$\begin{array}{c} \pi_{mr}^{RC}=\left(p_{r}-c-\alpha \delta \right)D_{r}^{RC}+\left(p_{m}-c-\alpha \delta \right)D_{m}^{RC}-\frac{\varphi }{2}\beta^{2}-\frac{A_{m}^{2}}{2}-\frac{A_{r}^{2}}{2}, \end{array}$$ (25)

According to the demand and profit function of each participant in the supply chain and the Stackelberg game order, theorem 3 can be obtained.

**Theorem 3:** In this part, the retailer first determines the optimal level of advertising and the optimal proportion of sharing, and then the supplier determines the optimal proportion of advertising and the optimal proportion of advertising cost sharing. We calculate retailer first, we can get the partial derivative of the advertising investment level and allocation proportion in the retailer’s profit function:

$$\begin{array}{c} \frac{\partial \pi_{r}^{RC}}{\partial A_{r}}=\left(1-\gamma \right)\left(p_{r}-\omega \right)K_{r}-\left(1-t_{m}\right)A_{r}, \end{array}$$ (26)

$$\begin{array}{c} \begin{array}{cc} \frac{\partial \pi_{r}^{RC}}{\partial t_{r}}= & c-\eta p_{m}-2\eta p_{r}+2p_{r}+3\eta \omega -3\omega +\alpha \delta +(c-\eta p_{m}\\ + & 2\eta p_{r}-2p_{r}-\eta \omega +\omega +\alpha \delta )t_{r}. \end{array} \end{array}$$ (27)

Make this function equal to 0, we can get the optimal of retailer’s advertising investment level and proportion of advertising are:

$$\begin{array}{c} A_{r}=\frac{\left(p_{r}-\omega \right)\left(1-\gamma \right)K_{r}}{1-t_{m}}. \end{array}$$ (28)

$$\begin{array}{c} t_{r}^{*}=\frac{\eta \left(p_{m}-\omega +2\left(p_{r}-\omega \right)\right)-2\left(p_{r}-\omega \right)+\left(\omega -c-\alpha \delta \right)}{\eta \left(\omega -p_{m}+2\left(p_{r}-\omega \right)\right)-2\left(p_{r}-\omega \right)-\left(\omega -c-\alpha \delta \right)}. \end{array}$$ (29)

Then we can get the advertising investment level and allocation proportion in the supplier profit function by partial derivation, detailed calculation process are:

$$\begin{array}{c} \frac{\partial \pi_{m}^{RC}}{\partial A_{m}}=\left(1-\eta \right)\left(\omega -c-\alpha \delta \right)K_{m}+\eta \left(p_{m}-c-\alpha \delta \right)K_{m}-\left(1-t_{r}\right)A_{m}, \end{array}$$ (30)

$$\begin{array}{c} \frac{\partial \pi_{m}^{RC}}{\partial t_{m}}=-\frac{A_{r}^{2}}{2}. \end{array}$$ (31)

Make this function equal to 0, we can get the optimal of supplier’s advertising investment level and proportion of advertising are:

$$\begin{array}{c} t_{m}^{*}=0, \end{array}$$ (32)

$$\begin{array}{c} A_{m}=\frac{\left(\eta \left(p_{m}-\omega \right)+\omega -c-\alpha \delta \right)K_{m}}{1-t_{r}}. \end{array}$$ (33)

Substituting $t_{r}^{*}$ into *A*_*m*_ and substituting $t_{m}^{*}$ into *A*_*r*_, we can get that $A_{m}^{*}$ and $A_{r}^{*}$ are:

$$\begin{array}{c} A_{m}^{*}=\frac{K_{m}}{2}\left(\eta \left(p_{m}-2p_{r}+\omega \right)+2p_{r}-\omega -c-\alpha \delta \right), \end{array}$$ (34)

$$\begin{array}{c} A_{r}^{*}=\left(1-\gamma \right)\left(p_{r}-\omega \right)K_{r}. \end{array}$$ (35)

## 5 Model analysis

### 5.1 Optimal decision analysis of cooperative advertising models

In the basic model, three cooperative advertising strategies and profits in the dual channel supply chain of fresh agricultural products are analyzed respectively, and there are still many factors that have a profound impact on cooperative advertising strategies, such as the level of advertising investment, the proportion of advertising cost sharing, and the efficiency of carbon emission reduction. Therefore, this section analyzes the impact of the above factors on the optimal decision of cooperative advertising strategies, as shown in the following propositions 1-4.

**Proposition 1:**

$$\begin{array}{c} \begin{array}{cc} & \frac{\partial A_{m}^{*}}{\partial p_{m}}=\frac{\eta K_{m}}{2}>0,\;\frac{\partial A_{m}^{*}}{\partial p_{r}}=\left(1-\eta \right)K_{m}>0,\\ & \frac{\partial A_{m}^{*}}{\partial \omega }=-\frac{K_{m}}{2}<0,\;\frac{\partial A_{m}^{*}}{\partial c}=-\frac{K_{m}}{2}<0. \end{array} \end{array}$$ (36)

Proposition 1 shows that in the *RM* model, with the increase of direct selling price, retail price, supplier will increase the level of advertising investment, that is when the price between two channels increases, supplier will continue to increase advertising investment to expand market demand, so that consumers can understand low-carbon fresh agricultural products more, and then improve sales and recognition of low-carbon fresh agricultural products. However, when the production cost of goods and the wholesale price of suppliers rise, supplier will reduce advertising. This is because the rise in commodity production costs will cause supplier to raise wholesale prices, which will eventually lead to the rise in commodity prices, and will also cause consumers to lose their enthusiasm for purchasing, which will have a negative impact on the profits of supply chain participants. Therefore, in order to ensure that the profit is not affected, the supplier will reduce the advertising investment.

**Proposition 2:**

$$\begin{array}{c} \frac{\partial t_{r}^{*}}{\partial p_{m}}=\frac{-4\eta \left(1-\eta \right)\left(p_{r}-\omega \right)}{{\left(\alpha \delta +c-\eta p_{m}+2\eta p_{r}-\eta \omega -2p_{r}+\omega \right)}^{2}}<0, \end{array}$$ (37)

$$\begin{array}{c} \frac{\partial t_{r}^{*}}{\partial p_{r}}=\frac{4\left(1-\eta \right)(\eta \left(p_{m}-\omega \right)+\omega -c-\alpha \delta )}{{\left(\alpha \delta +c-\eta p_{m}+2\eta p_{r}-\eta \omega -2p_{r}+\omega \right)}^{2}}>0, \end{array}$$ (38)

$$\begin{array}{c} \frac{\partial t_{r}^{*}}{\partial \omega }=\frac{4\left(1-\eta \right)(\eta \left(p_{r}-p_{m}\right)-p_{r}+c+\alpha \delta )}{{\left(\alpha \delta +c-\eta p_{m}+2\eta p_{r}-\eta \omega -2p_{r}+\omega \right)}^{2}}<0, \end{array}$$ (39)

$$\begin{array}{c} \frac{\partial t_{r}^{*}}{\partial c}=\frac{4\left(1-\eta \right)\left(p_{r}-\omega \right)}{{\left(\alpha \delta +c-\eta p_{m}+2\eta p_{r}-\eta \omega -2p_{r}+\omega \right)}^{2}}>0, \end{array}$$ (40)

$$\begin{array}{c} \frac{\partial t_{r}^{*}}{\partial \alpha }=\frac{4\delta \left(1-\eta \right)\left(p_{r}-\omega \right)}{{\left(\alpha \delta +c-\eta p_{m}+2\eta p_{r}-\eta \omega -2p_{r}+\omega \right)}^{2}}>0. \end{array}$$ (41)

Proposition 2 shows that in the *RM* model, when the direct selling price and wholesale price of supplier increases, retailer will reduce the advertising cost of supplier, while when retailer increases the selling price and commodity cost price, they will increase the investment in the proportion of advertising cost. This shows that when the direct selling price of supplier rises, it will increase the profits of supplier, but has nagetive impact on the profits of retailer. According to the “rational person” hypothesis, retailer will reduce the proportion of sharing the advertising costs of supplier. In addition, when supplier increases the wholesale price of products, the marketing cost of retailer increases, so retailer choose to reduce the proportion of advertising cost sharing to protect own profit. When the retailer’s selling price increases, the retailer chooses to increase its share of the supplier’s advertising cost due to the increase of profit. When the carbon emission reduction efficiency increases, that is, the green degree of fresh agricultural products is increased, retailer will also be more active in promoting low-carbon fresh agricultural products; While increasing the efficiency of carbon emission reduction will also increase the production cost of goods, retailer will improve the publicity of low-carbon fresh agricultural products, so that more consumers can understand low-carbon fresh agricultural products.

**Proposition 3:**

$$\begin{array}{c} \frac{\partial A_{r}^{*}}{\partial p_{r}}=\left(1-\gamma \right)K_{r}>0,\;\frac{\partial A_{r}^{*}}{\partial \omega }=-\left(1-\gamma \right)K_{r}<0. \end{array}$$ (42)

Proposition 3 shows that in the *RR* model, with the rise of wholesale prices, retailer will reduce the level of advertising. Because when the wholesale price rises, which will have a negative impact on the profits of retailer, so retailer will also reduce the level of advertising investment. But when the retail price rises, retailer benefts form it and will increase investment in advertising.

**Proposition 4:**

$$\begin{array}{c} \begin{array}{cc} & \frac{\partial A_{m}}{\partial p_{m}}=\frac{\eta K_{m}}{2}>0,\;\frac{\partial A_{m}}{\partial p_{r}}=K_{m}>0,\\ & \frac{\partial A_{m}}{\partial \omega }=-\frac{K_{m}}{2}\left(1-\eta \right)<0,\;\frac{\partial A_{m}}{\partial c}=-\frac{K_{m}}{2}<0. \end{array} \end{array}$$ (43)

$$\begin{array}{c} \frac{\partial A_{r}}{\partial p_{r}}=\left(1-\gamma \right)K_{r}>0,\;\frac{\partial A_{r}}{\partial \omega }=-\left(1-\gamma \right)K_{r}<0. \end{array}$$ (44)

Proposition 4 shows that in the *RC* model, with the increase of the price of supplier and retailer, both sides are willing to increase advertising investment to expand market demand. When the wholesale price rises, both supplier and retailer will reduce the level of advertising investment. In addition, when the production cost of low-carbon fresh agricultural products rises, supplier will have less advertising investment. When the production cost of goods rises, the selling price of both channels will increase, because the offline channels of fresh agricultural products have a better sense of experience and gain, so the online direct selling is damaged more obviously, which leads to the reduction of advertising investment by supplier. Due to the increase in the cost of producing low-carbon fresh agricultural products will lead to the rise in the wholesale price of commodities and the decline in market demand, then affect the profit of supplier, thus supplier will reduce the investment of supplier in advertising.

### 5.2 Comparative analysis of cooperative advertising models

Further, we make a comparative analysis of the three cooperative advertising strategies in this section, by comparing the differences between the demands of online and offline sales channels and the profit levels of all parties in different cooperative advertising models, it can be seen in propositions 5 and 6.

**Proposition 5:**

$$\begin{array}{c} D_{m}^{RC}-D_{m}^{RM}=K_{r}^{2}\gamma \left(1-\gamma \right)\left(p_{r}-\omega \right)>0, \end{array}$$ (45)

$$\begin{array}{c} D_{m}^{RC}-D_{m}^{RR}=\frac{\eta K_{m}^{2}}{2}\left(\eta \left(p_{m}-2p_{r}+\omega \right)+2p_{r}-\omega -c-\alpha \delta \right)>0, \end{array}$$ (46)

$$\begin{array}{c} D_{r}^{RC}-D_{r}^{RM}=K_{r}^{2}{\left(1-\gamma \right)}^{2}\left(p_{r}-\omega \right)>0, \end{array}$$ (47)

$$\begin{array}{c} D_{r}^{RC}-D_{r}^{RR}=\frac{\left(1-\eta \right)K_{m}^{2}}{2}\left(\eta \left(p_{m}-2p_{r}+\omega \right)+2p_{r}-\omega -c-\alpha \delta \right)>0. \end{array}$$ (48)

Proposition 5 shows that from the perspective of online and offline demand, the situation of two-way cooperative advertising between supplier and retailer is better than that of one-way cooperative advertising between supplier and retailer. In the *RC* model, the two sides jointly invest in cooperative advertising, which is more conducive to promoting the increase of market demand and expanding the market scale of fresh agricultural products.

**Proposition 6:**

$$\begin{array}{c} \pi_{m}^{RC}-\pi_{m}^{RM}=K_{r}^{2}\left(p_{r}-\omega \right)\left(1-\gamma \right)\left(p_{m}\gamma -\omega \gamma +\omega -c-\alpha \delta \right)>0, \end{array}$$ (49)

$$\begin{array}{c} \pi_{m}^{RC}-\pi_{m}^{RR}=\frac{K_{m}^{2}}{4}\left(\eta p_{m}-\eta \omega +\omega -c-\alpha \delta \right)\left(\eta p_{m}-2\eta p_{r}+2p_{r}+\eta \omega -\omega -c-\alpha \delta \right)>0, \end{array}$$ (50)

$$\begin{array}{c} \pi_{r}^{RC}-\pi_{r}^{RM}=\frac{K_{r}^{2}}{2}{\left(p_{r}-\omega \right)}^{2}{\left(1-\gamma \right)}^{2}>0, \end{array}$$ (51)

$$\begin{array}{c} \pi_{r}^{RC}-\pi_{r}^{RR}=\frac{K_{m}^{2}}{8}{\left(\eta p_{m}-2\eta p_{r}+2p_{r}+\eta \omega -\omega -c-\alpha \delta \right)}^{2}>0. \end{array}$$ (52)

Proposition 6 shows that comparing the profits of supplier and retailer under different cooperative advertising strategies, it can be seen that two-way cooperative advertising between supplier and retailer is superior to the other two models. In the *RC* model, supplier and retailer can obtain higher profits. Through two-way investment and cooperative advertising, the profit level of both sides can be significantly improved, laying the foundation for the sustainable development of the dual channel supply chain of fresh agricultural products.

## 6 Numerical analysis

Based on the above theoretical analysis, in order to more intuitively reflect the relationship between various influencing factors and profits, channel demand and cooperative advertising strategy, this section respectively analyzes the *RM* model, *RR* model and *RC* model by calculating examples. Parameters for the impact of advertising on demand according to literature [51], parameters for consumer acceptance of suppliers’ online direct sales channels according to literature [42], and parameters related to carbon reduction according to literature [15], and we assign values to the other parameters according to the assumed conditions in the paper, so the parameters are set as follows in Table 3:

**Table 3:** Design of relevant parameters.

*θ*	*T*	*η*	*γ*	*δ*	*φ*	*μ*	λ	*K*_*m*_	*K*_*r*_	*p*_*m*_	*p*_*r*_	*ω*	*c*	*α*	*β*
0.5	100	0.7	0.3	0.5	1	1	1	1	1	15	17	10	5	1	1

**Fig 3:**
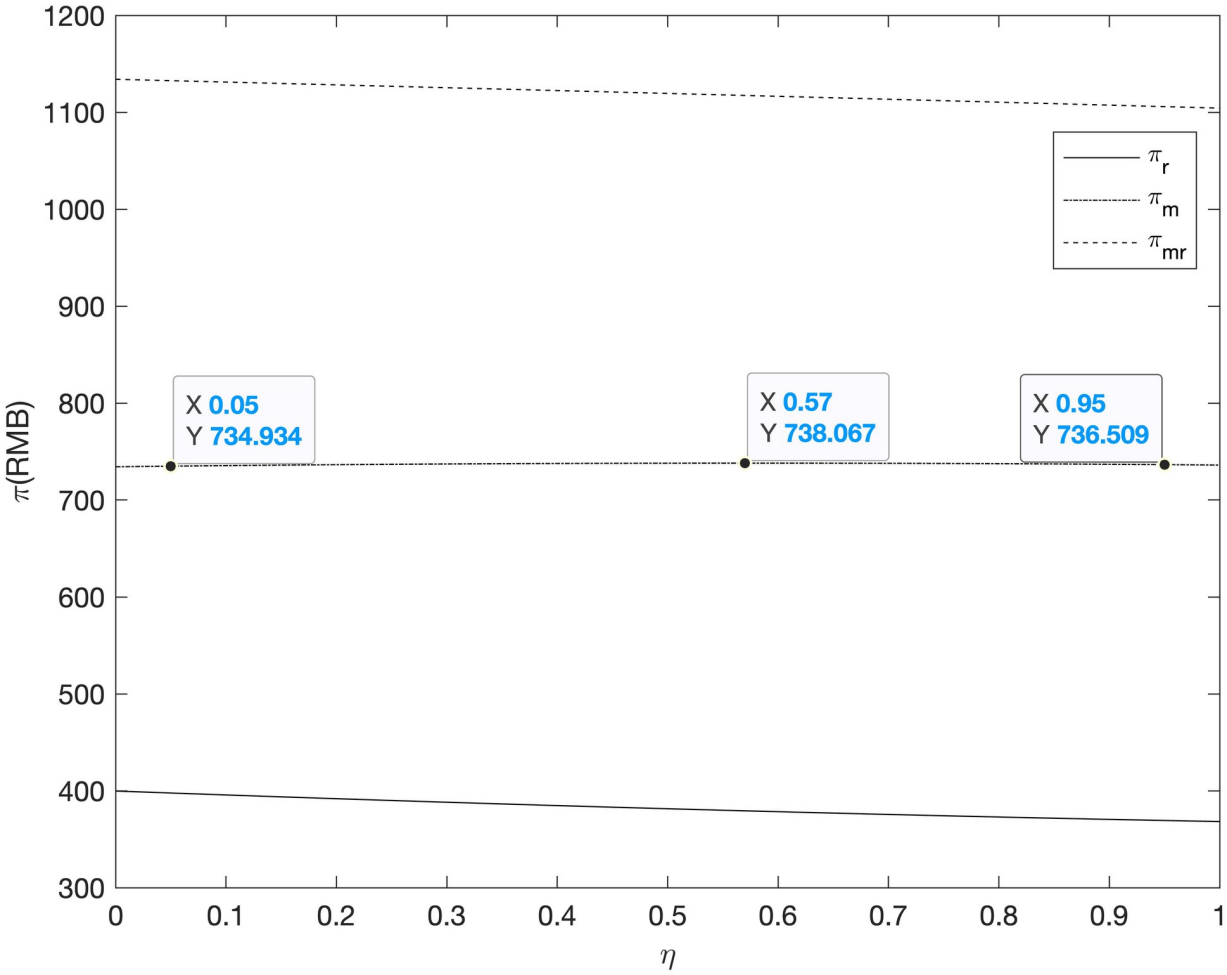
The relationship between profits $\pi_{m}^{RM}/\pi_{r}^{RM}/\pi_{mr}^{RM}$ and *η* of supply chain members (*RM*).

**Fig 4:**
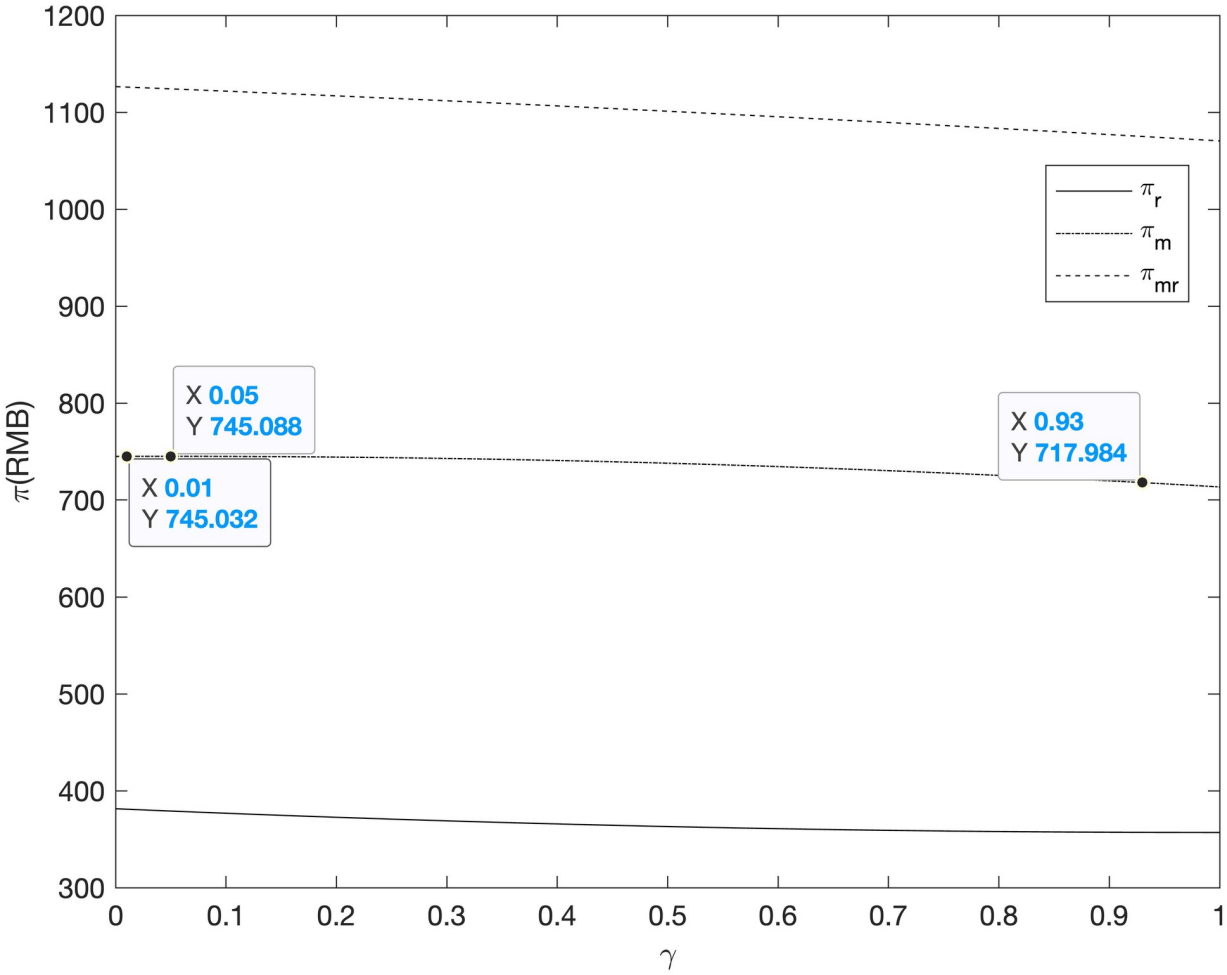
The relationship between profits $\pi_{m}^{RR}/\pi_{r}^{RR}/\pi_{mr}^{RR}$ and *γ* of supply chain members (*RR*).

**Fig 5:**
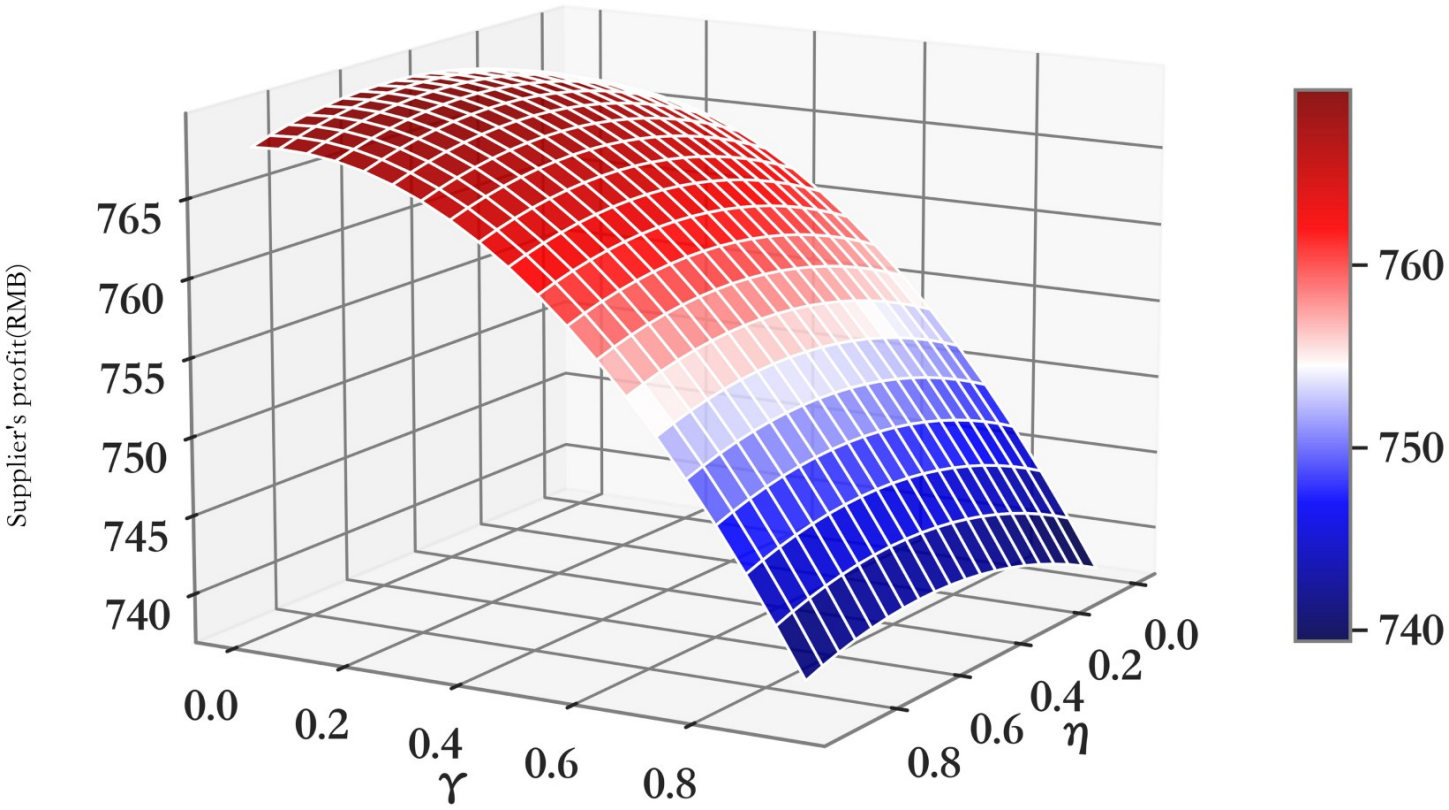
The relationship between profit $\pi_{m}^{RC}$ and *η*/*γ* of supply chain members (*RC*).

**Fig 6:**
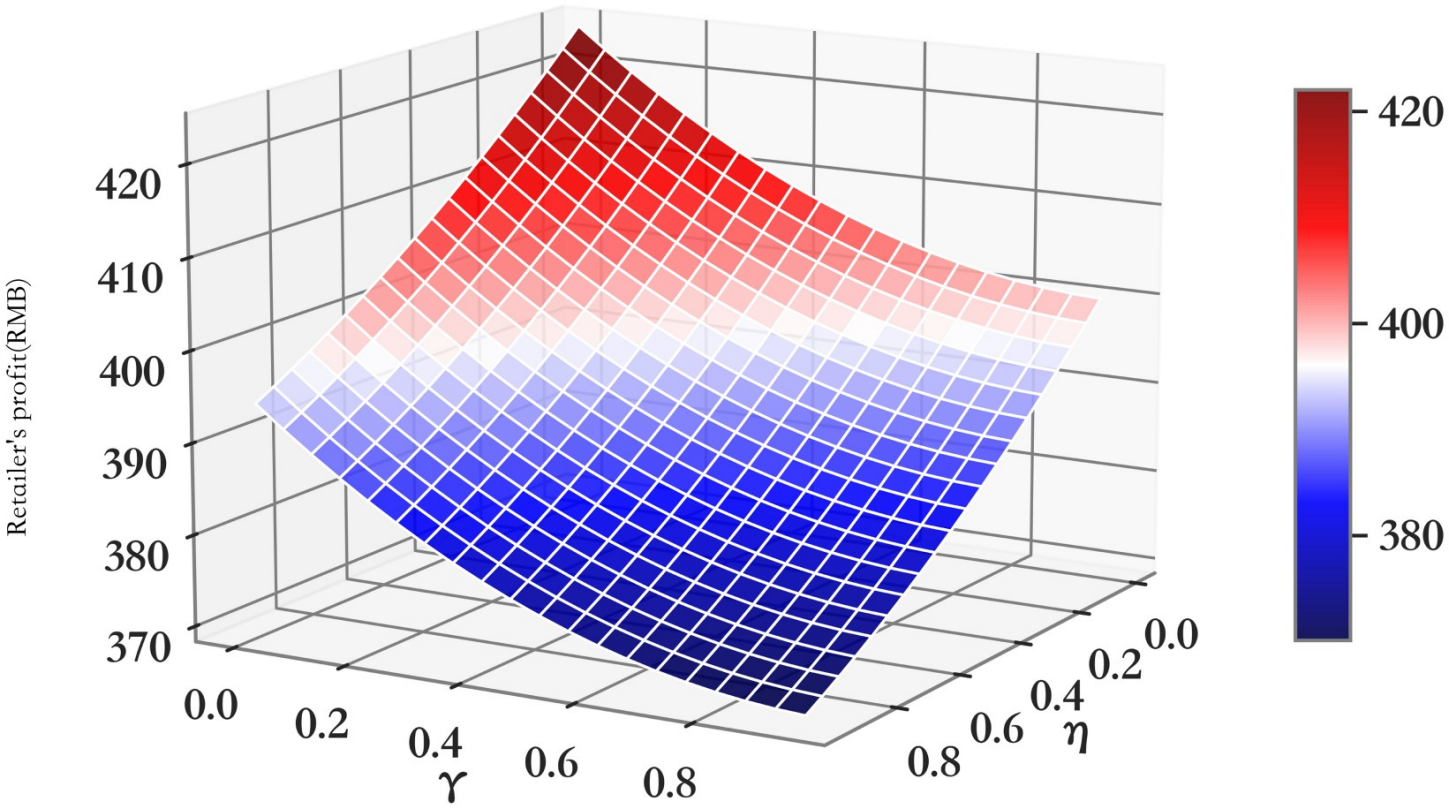
The relationship between profit $\pi_{r}^{RC}$ and *η*/*γ* of supply chain members (*RC*).

**Fig 7:**
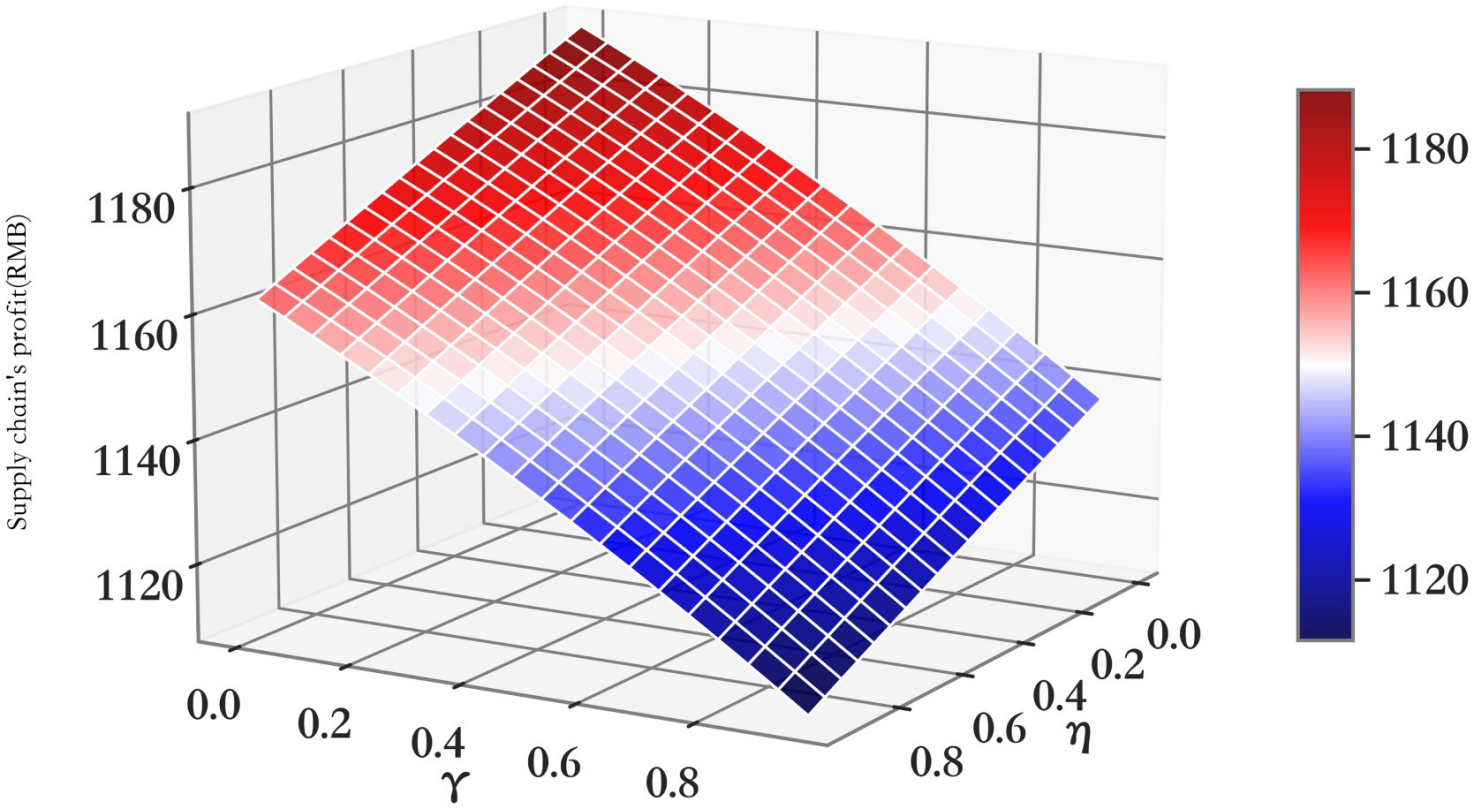
The relationship between profit $\pi_{mr}^{RC}$ and *η*/*γ* of supply chain members (*RC*).

First of all, among the three cooperative advertising modes, because the profit function model of supplier, retailer and the supply chain as a whole is more complex, this section analyzes the impact of the coefficient of advertising investment on online and offline demand on the profits of supplier, retailer and the supply chain. The results are shown in Figs 3–7.

It can be seen from Figs 3 and 4 that in the *RM* and *RR* model, with the increase of the impact of cooperative advertising on online and offline demand, the profit of supplier increased first and then decreased, while the profit of retailer decreased, which led to the decline of the overall profit of the supply chain. As this paper analyzes the supplier independent advertising situation, with the increasing impact of cooperative advertising on the demand between two channels, supplier will benefit to a certain extent, while retailer’s profit will be damaged due to the increase in expenditure.

It can be seen from Figs 5–7 that with the increase of the impact of cooperative advertising on online and offline demand, the profit of supplier first increased and then decreased, the profit of retailer shows a downward trend, but with the deepening impact of advertising on demand, the loss of profit will be alleviated to some extent. Due to the obvious decline in the profits of retailers, the overall profits of the supply chain have been declining. However, by comparing the three cooperative advertising modes, it can be seen that the overall profits of supplier, retailer and supply chain are the highest in the mode. The profit level of supply chain participants can be effectively increased through the launch of two-way cooperative advertising.

**Fig 8:**
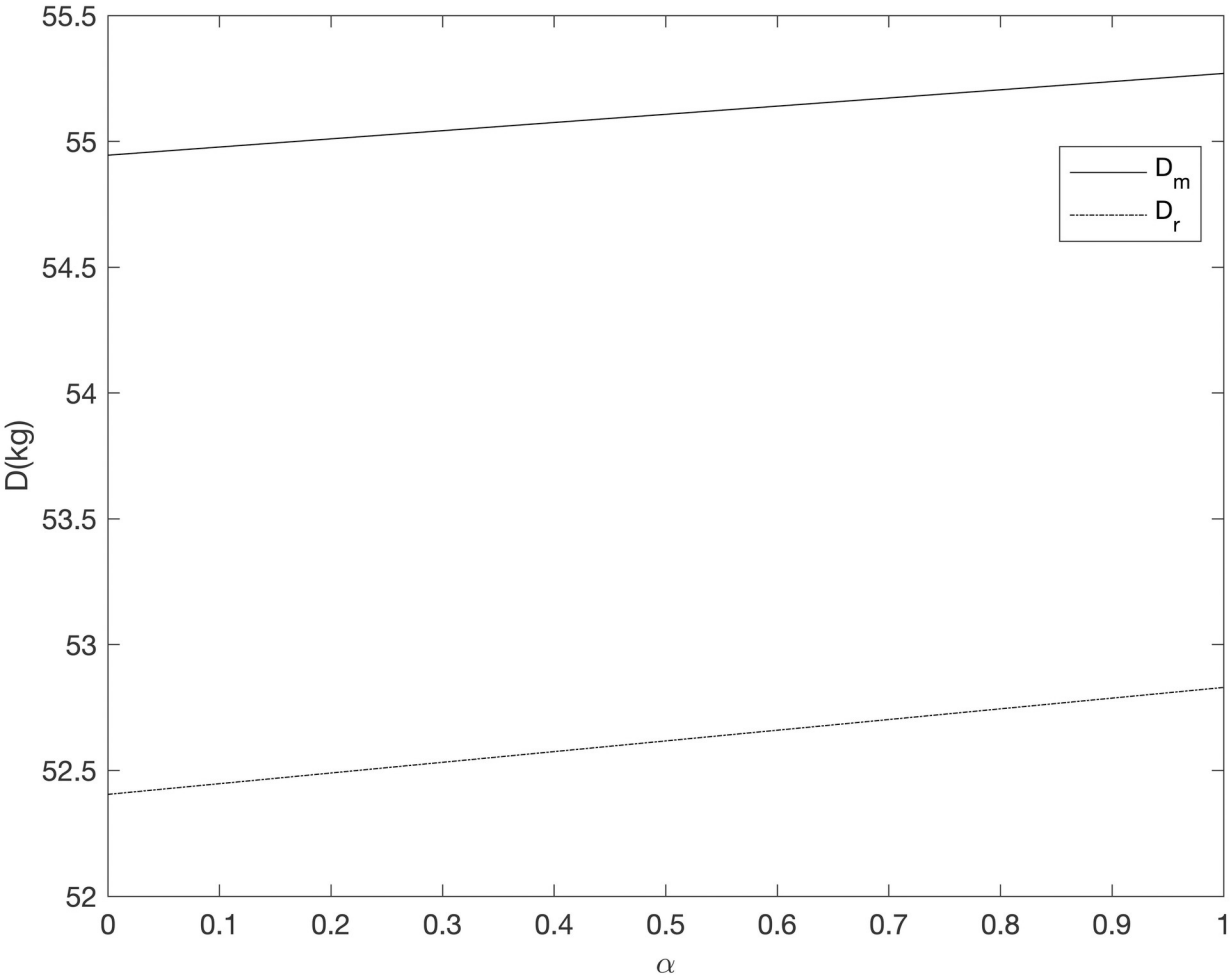
The relationship between dual channel demand and *α* (*RM*).

**Fig 9:**
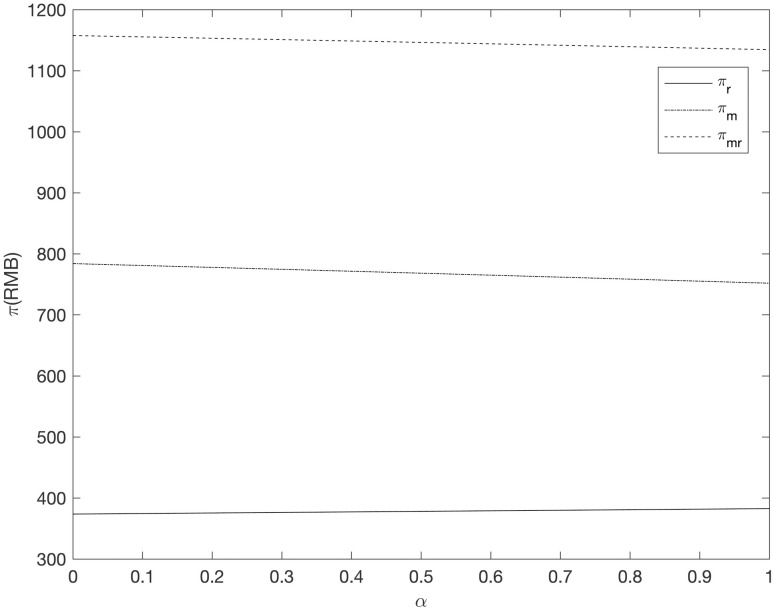
The relationship between supply chain members’ profit and *α* (*RM*).

**Fig 10:**
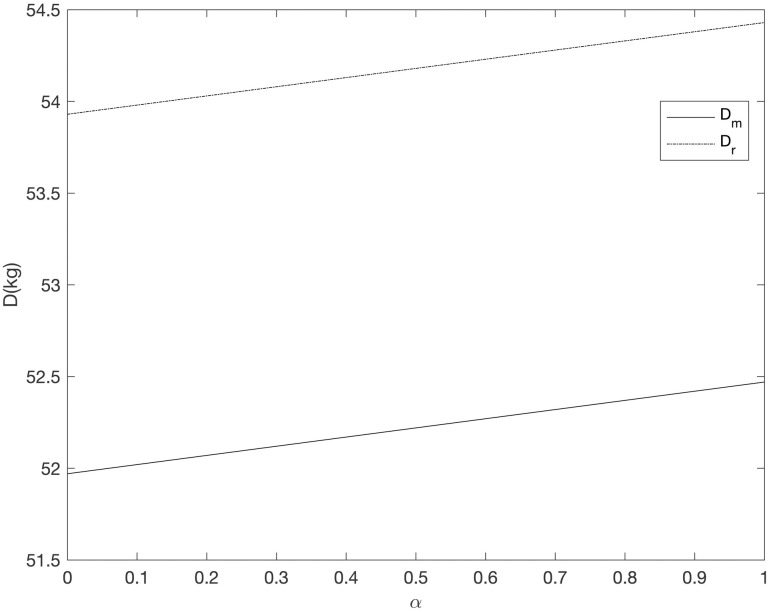
The relationship between dual channel demand and *α* (*RR*).

**Fig 11:**
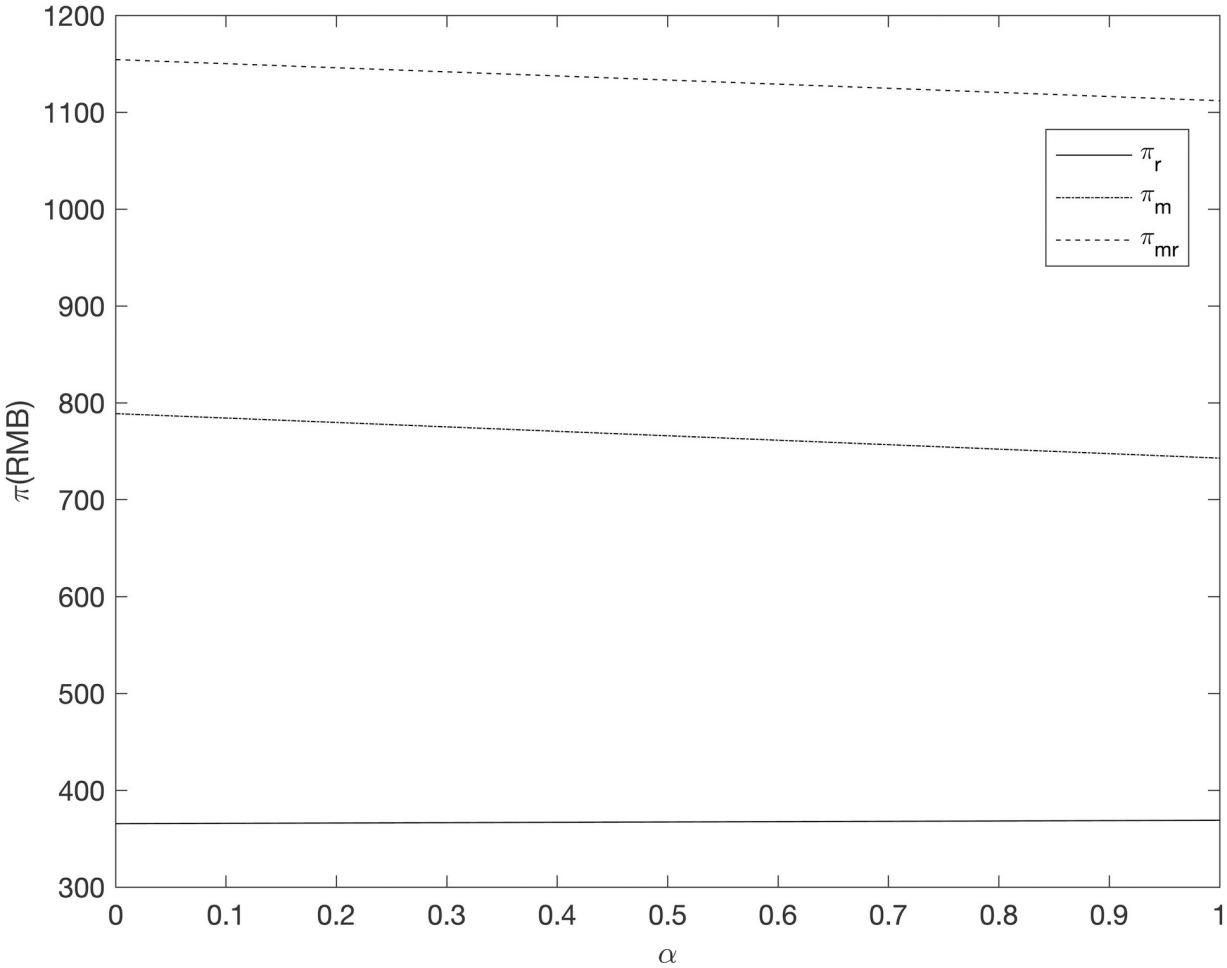
The relationship between supply chain members’ profit and *α* (*RR*).

**Fig 12:**
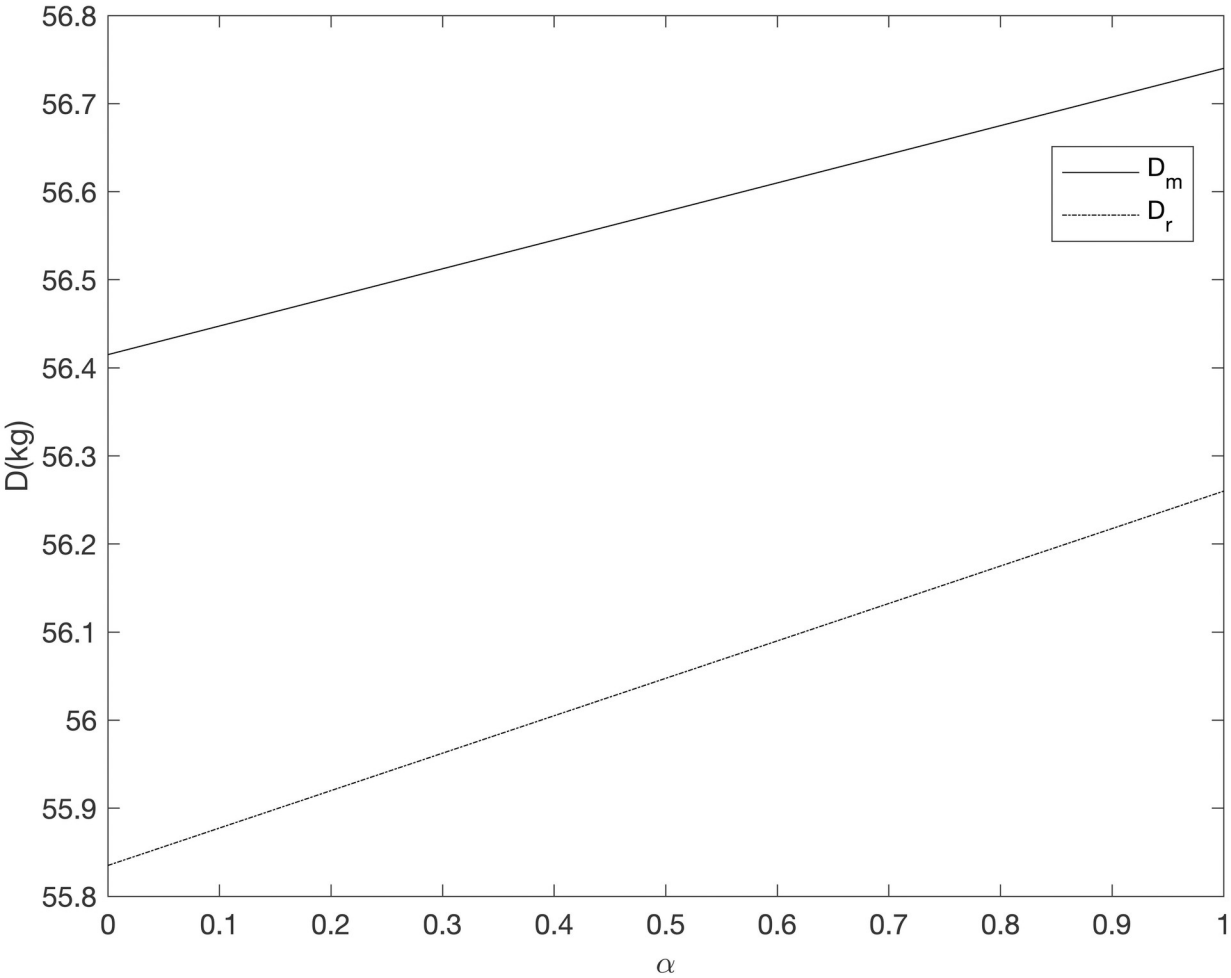
The relationship between dual channel demand and *α* (*RC*).

**Fig 13:**
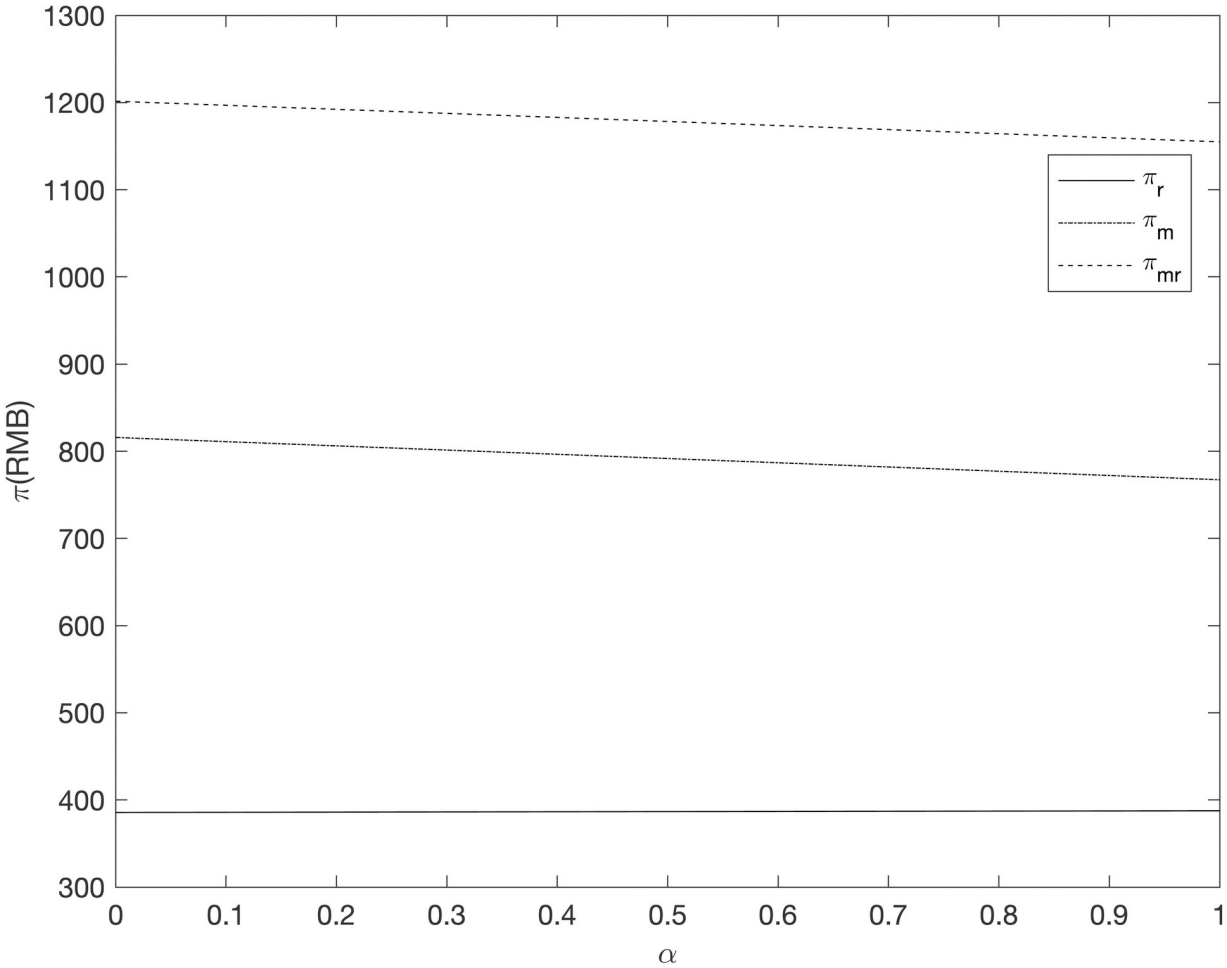
The relationship between supply chain members’ profit and *α* (*RC*).

It can be seen from Figs 8–13 that in the three cooperative advertising modes, with the increase of carbon emission reduction efficiency, the demand of online and offline dual channels has shown a significant upward trend, and the growth of offline demand of retailer is greater than that of supplier. Compared with the other two cooperative advertising modes, the demand growth of dual channels is the most significant in the *RC* model. Although consumers’ demand for low-carbon fresh agricultural products has increased, the investment cost of carbon emission reduction technology and the publicity cost of low-carbon fresh agricultural products are still expenses that can not be ignored by supplier and retailer, resulting in a downward trend in supplier’s profit and a slight upward trend in retailer’s profit. By comparing the three cooperative advertising modes, it can be seen that supplier, retailer and the supply chain as a whole all reap the highest profits in the *RC* model; In the *RR* model, the profit of retailers increases the fastest, while the profit of supplier and supply chain as a whole decreases the least in the *RM* model.

## 7 Conclusion

### 7.1 Findings

Under the low-carbon development environment, this paper fully considers the situation that retailer dominate the dual channel supply chain of fresh agricultural products (an online direct selling supplier and an offline selling retailer), and provides help for the selection of cooperative advertising strategies in the supply chain of low-carbon fresh agricultural products by comparing the profit differences of each participant in different cooperative advertising strategies (three strategies include *RM*, *RR*, *RC*). It also analyzes the impact of carbon emission reduction efficiency, cost sharing, advertising investment level on the profit level of supply chain participants and cooperative advertising strategy selection. The research shows that:

1. Among the two one-way cooperative advertising models, retailer obtains higher profit in the *RM* model because retailer takes the initiative in the market and supplier increases consumers’ enthusiasm for purchasing through advertising, which leads to the most significant increase in retailer’s profit. In the *RR* model, retailer can obtain higher market share, which due to the retailer increases consumers’ offline shopping demand through advertising, but due to the increase of marketing costs caused by the retailer’s advertisements, which makes the retailers’s profit level in the *RM* model higher than the *RR* model. And by observing the two one-way cooperative advertising models, it can be seen that both supplier and retailer placing advertisements will increase consumers’ interest in the channel and benefit channel sales.
2. In the *RC* model, the overall profits of supplier, retailer and supply chain are not the highest among the three models, which are significantly higher than the other two one-way cooperative advertising models (*RM*/*RR*). In addition, as the impact of cooperative advertising on demand deepens, the demand of supplier’s online channel and retailer’s offline channel will gradually increase, providing practical support and help for the further development of the dual channel supply chain of fresh agricultural products. Combined with the example analysis, it can be seen that the participants in the supply chain should control the level of advertising investment to ensure the profit level of each participant.
3. Under the background of the initiative of global low-carbon development and the strengthening of consumers’ concept of environmental protection, when the efficiency of carbon emission reduction gradually increases, the market demand in the three cooperative advertising modes gradually increases, which shows that consumers are willing to consume low-carbon fresh agricultural products, indicating that the cooperative advertising strategy involved in the dual channel supply chain of fresh agricultural products has laid a good foundation. However, only the profit level of supplier increases with the increase of carbon emission reduction efficiency, while the profit level of retailer declines periodically due to the high cost of investment in cooperative advertising.

### 7.2 Implications

This study has important practical significances on the choice of cooperative advertising mode, expanding market demand and increasing profit for fresh agricultural products dual-channel supply chain, and we make two implications to suppliers and retailers:

First, when supplier and retailer simultaneously invest cooperative advertisements (*RC*), supplier, retalier and the supply chain have optimal profits, and by publicizing fresh agricultural products with carbon emission reduction technologies in both directions, we can better increase consumers’ trust and recognition, promote the rise of market demand for fresh agricultural products, and provide a strong impetus for the development of a low-carbon economy.
Second, by sharing each other’s advertising costs, free-riding behavior is effectively avoided, so that all participants in the supply chain contribute to the common development. Low-carbon development is a general trend, through the input of cooperative advertising, to determine the optimal profit under different cooperative advertising modes, to provide practical help for the future strategic choices of supplier and retailer.

### 7.3 Limitation and future research

This paper still has some limitations. When supplier is unwilling to bear the advertising costs of retailer, they should introduce relevant contracts to coordinate the profits, and reduce the possibility of supplier’s free riding behavior. On the basis of this paper, the subsequent research can study the cooperative advertising strategy of the dual channel supply chain of low-carbon fresh agricultural products from the perspective of centralized decision-making. With the advent of the big data era, algorithms such as mechanical learning, digital twin and other technologies and methods can be integrated into the research of low-carbon supply chains in the future to accelerate the development of carbon emission reduction.

## Supporting information

S1 File (ZIP)
