# Correction: Research on cooperative advertising strategies for dual channel supply chain of fresh agricultural products considering carbon reduction efficiency under retailer leadership

**DOI:** 10.1371/journal.pone.0310261

**Published:** 2024-09-06

**Authors:** Wenbo Wang, Aimin Zhu, Lijuan Yu, Hongjiang Wei

The fourth author’s name is spelled incorrectly. The correct name is: Hongjiang Wei. The correct citation is: Wang W, Zhu A, Yu L, Wei H (2024) Research on cooperative advertising strategies for dual channel supply chain of fresh agricultural products considering carbon reduction efficiency under retailer leadership. PLoS ONE 19(6): e0303525. https://doi.org/10.1371/journal.pone.0303525
